# Private Nurses and the College

**Published:** 1920-11-13

**Authors:** 


### THE MATRONS' AND SISTERS' DEPARTMENT.

### PRIVATE NURSES AND THE COLLEGE.

There are very few nurses who do not at one time or another in the course of their career undertake the care of the sick in their own houses. If ever accurate statistics of the nursing profession are procurable it is safe to prophesy that by far the largest section of nurses would be found in the ranks of private nursing. Hence it is of the utmost importance that this part of the profession should find itself. It does not suffice that it is very largely represented in the ranks of College members. They should stand shoulder to shoulder and be in a position to form a collective judgment on the things which concern their interests as distinct from the interests of other women in the profession.

Private nursing gathers into its fold many varieties of nursing sisters, according to the special maladies they have been trained to deal with. While there is a general consensus of opinion that none are worthy the name of nurse who have not studied in a fair-sized general hospital, there is still room for many distinctions according to the qualifications grafted on, before or after, the general training. The private nursing section would unite mental, fever, children's, obstetric, and many other varieties of subtly specialising nurses, by a common bond of interest. Would it not be possible for private nursing members of the College to form a community within a community, owning their local centre as the point of contact, and drawing the cords of mutual association for a common purpose closer by intercommunication between other centres?

There are many clubs and societies in which unity of purpose is not found incompatible with diversity of operations. To be a member of the College is good and very good. Suppose the member being a private nurse desires also to make one of a body of private nurses who will look at things from her own stand-point, should it not be possible for her to find this body ready to admit her among themselves in her College centre? The advantages which would accrue to her by such a subdivision would be numerous. In the first place the tie which binds her to the College would be no wise weakened, but infinitely strengthened by finding out those of her fellow members who like herself are private nurses. She comes, perhaps, as a stranger among the other members, and it would help her to feel that the Central Body existed for her benefit as well as for the rest, if she found other birds of passage among them, and was welcomed to the ranks of her own section. Next, the formation of a special section for private nurses would give opportunity for a little more healthy self-government. Meetings might be arranged to suit the varying convenience of members of this section, attention would be drawn to their requirements in club-rooms, etc., especially when on night duty, and some important aspects of their branch of the profession, such as the keeping of business accounts, might be handled by experts.

But perhaps the most vital matter in rallying private nurses within the College relates to elections. It is solely by bringing particular classes of members together under the ægis of the College and setting them to manage their own affairs that a right understanding can be attained about the persons to vote for and the things which it is desired the Council should undertake. If every local centre had its own group of private nurses and all these groups were in correspondence with each other and met at given centres occasionally, there would no longer be any difficulty in getting really representative women to safeguard their special interests on the Council.

Strength in association is attained through the path of recognising diversity no less than through that of realising unity. The first step towards unity has been nobly attained. When the numbers touch the twentieth thousand it will be time to begin developing and sorting out, as it were, this great body of nursing sisters under their respective banners. So long as any one section believes itself in danger of being swamped or neglected, there is danger. Diversity is the badge of the nursing profession. The work undertaken by nurses differs to an incredible extent. Their aim is one.

So far, attention has concentrated with enormous success on the territorial divisions which separate nurses from each other and lend their interests a different colour in each locality. This has not broken the unity of the College, it has largely enhanced it. Perhaps in the future room may be found for another form of subdivision, subordinate to the successful localisation now achieved. In one part of the country after another we may see groups of members forming to discuss and draw together under the banner of public-health work, of institutional treatment and education, of private nursing, of many other branches. Of one thing we may be sure—that such an attempt at re-classification would call out new powers of development without sapping those which have been already so abundantly evidenced.

